# Protocol for a Phase Two, Parallel Three-Armed Non-inferiority Randomized Controlled Trial of Acceptance and Commitment Therapy (ACT-Adjust) Comparing Face-to-Face and Video Conferencing Delivery to Individuals With Traumatic Brain Injury Experiencing Psychological Distress

**DOI:** 10.3389/fpsyg.2021.652323

**Published:** 2021-03-08

**Authors:** Diane L. Whiting, Grahame K. Simpson, Frank P. Deane, Sarah L. Chuah, Michelle Maitz, Jerre Weaver

**Affiliations:** ^1^Brain Injury Rehabilitation Research Group, Ingham Institute for Applied Medical Research, Liverpool, NSW, Australia; ^2^School of Psychology, University of Wollongong, Wollongong, NSW, Australia; ^3^John Walsh Centre for Rehabilitation Research, Sydney School of Medicine, University of Sydney, St Leonards, NSW, Australia; ^4^Liverpool Brain Injury Rehabilitation Unit, Liverpool Hospital, Liverpool, NSW, Australia; ^5^Mid-Western Brain Injury Rehabilitation Unit, Bathurst, NSW, Australia

**Keywords:** traumatic brain injury, acceptance and commitment therapy, video conferencing, randomized control trial, telehealth, psychological distress

## Abstract

**Background:** People with traumatic brain injury (TBI) face a range of mental health challenges during the adjustment process post-injury, but access to treatment can be difficult, particularly for those who live in regional and remote regions. eHealth provides the potential to improve access to evidence-based psychological therapy for people with a severe TBI. The aim of the current study is to assess the efficacy of a psychological intervention delivered via video consulting to reduce psychological distress in people with TBI.

**Methods:** This paper outlines the protocol for a multi-center, three-arm, parallel, non-inferiority randomized controlled trial (RCT) of an evidence-based manualized psychological intervention, ACT-Adjust. ACT-Adjust provides nine sessions for adults with a moderate to severe TBI experiencing clinical levels of psychological distress. Fifty-six participants referred from Brain Injury Rehabilitation Units across New South Wales (NSW) and the NSW icare scheme will be randomly allocated to three conditions; (1) video consulting (VC), (2) face-to-face (FtF) and, (3) a waitlist control (WL).

**Discussion:** This is the first RCT to evaluate the efficacy of a psychological therapy (ACT-Adjust) delivered via video consulting for individuals with a moderate to severe TBI.

**Trial Registration:** www.anzctr.org.au, Australian New Zealand Clinical Trials Registry ANZCTRN2619001602112.

## Introduction

Psychological distress following a traumatic brain injury (TBI) is highly prevalent (Gould et al., 2011) and includes mixed mental health problems with features of depression, anxiety, anger, and stress. Whilst first line treatment often involves medication aimed at symptom reduction, this alone does not provide the individual with strategies to address the complex psychological adjustment process following TBI. Psychological interventions utilizing cognitive behavioral therapy (CBT) to ameliorate mental health symptomology tend to be disorder-specific, targeting specific symptoms of hopelessness, anxiety, depression, and anger (Medd and Tate, 2000; Simpson et al., 2011; Hsieh et al., 2012; Fann et al., 2015). Acceptance and Commitment Therapy (ACT) is a transdiagnostic treatment with the therapeutic goal of assisting people in managing their mental health through increased psychological flexibility.

ACT encompasses the six core processes of acceptance, defusion, contact with the present moment, self as context, values identification, and committed action with the central aim of increasing psychological flexibility. Acceptance involves noticing unpleasant or unwanted internal experiences without trying to change or avoid them, while defusion uses a range of experiential exercises to assist in changing the relationship with those unpleasant internal experiences. Being in contact with the present moment allows the person to be aware without judgment and without influencing or changing the experience. Self as context or the observing self, allows the person to see themselves as separate from their thoughts, feelings, and experiences and notice there is a transcendent self that remains the same. Mindfulness exercises are used to assist movement through these processes. Values and committed action constitute the behavioral component of ACT and allows the individual to focus their behavior and set goals in a way that is consistent with their underlying principles. ACT uses experiential exercises and metaphors which can be adapted and applied to individuals who have a cognitive impairment as a result of a TBI (Whiting et al., 2017).

ACT-Adjust is an evidence-based treatment program incorporating the transdiagnostic components of ACT with modifications to account for the cognitive impairments experienced by individuals with a severe TBI (Whiting et al., 2012). It has been specifically designed to facilitate psychological adjustment after a severe TBI. In a recent clinical trial testing the feasibility of the program, ACT-Adjust achieved both statistically and clinically significant reductions in levels of depression and stress amongst clients with a moderate to severe TBI, compared to an active control group (Whiting et al., 2020).

Despite the growing evidence-base for interventions treating psychological distress following a TBI (Medd and Tate, 2000; Simpson et al., 2011; Hsieh et al., 2012; Fann et al., 2015; Ponsford et al., 2016; Abel et al., 2017; Brenner et al., 2018; Dindo et al., 2020; Whiting et al., 2020), access to psychological services is limited for regional and remote people across Australia (Bourne et al., 2017), resulting in high levels of unmet needs (Simpson et al., 2016). eHealth, the provision of healthcare at a distance via technology, has the potential to increase access to services and has seen burgeoning research with a growing evidence base (Backhaus et al., 2012). ACT, for the treatment of a variety of mental health issues, has been delivered in a number of eHealth formats, including online, smartphone applications, and telehealth with positive outcomes (O'Connor et al., 2018). Within the TBI field, reviews have indicated positive effects of eHealth in supporting family members (Rietdijk et al., 2012) and in the provision of interdisciplinary rehabilitation care (Tran et al., 2017; Ownsworth et al., 2020). Although research related to use of eHealth delivery of interventions for people with TBI is limited, one case study design (*n* = 3) found delivery of treatment to address executive functioning via video was feasible on outcome measures of function and satisfaction with delivery method (Ng et al., 2013). Psychological therapy delivered by eHealth (telepsychology) for emotional disorders after a brain injury, however, has been limited to telephone-based counseling and has produced mixed results (Bell et al., 2005, 2011; Bombardier et al., 2009). An initial study comparing telephone delivery of a psychological intervention (motivational interviewing and solution-focused counseling) compared to usual care found significant differences between the groups, where the active condition demonstrated improved functional status and well-being (Bell et al., 2005), however when the study was replicated with a large sample (*n* = 433), no differences were found between the two groups (Bell et al., 2011).

To date, there is no research looking at the efficacy of psychological therapy to treat mental health issues delivered by video consulting to adults with a moderate to severe TBI. The purpose of this paper is to describe the design of a non-inferiority, randomized controlled trial, to examine whether an eHealth-based delivery of the ACT-Adjust treatment program achieves the same treatment effects as face-to-face delivery. It also will examine the efficacy of ACT-Adjust when compared to a wait-list control group and determine whether treatment gains are sustained three months after the intervention.

## Methods and Analysis

### Overview of Study Design and Research Hypotheses

**Figure 1:**
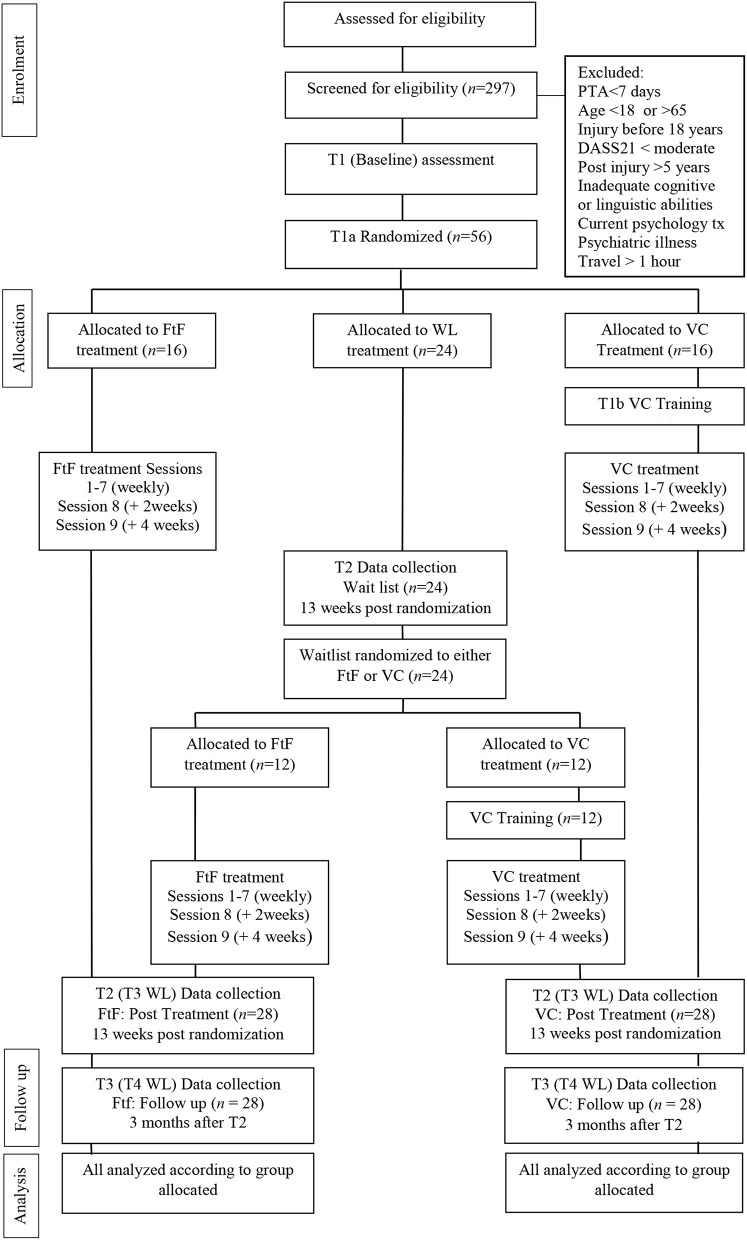
Study flow diagram.

The ACT-Adjust project aims to build on the success of the original feasibility trial (Whiting et al., 2020) and evaluate the benefits of utilizing eHealth for delivery of a psychological intervention to people with a TBI. This pre-registered, multi-center, three-arm, parallel, non-inferiority, single-blind randomized controlled trial (RCT) will test the efficacy of two modes of delivery of the ACT-Adjust intervention; face-to-face (FtF) and video consulting (VC), in comparison to a waitlist control group (see Figure 1).

This paper describes the study protocol (Dated September 30, 2019; Protocol Version 1.0) to comply with the Standard Protocol Items: Recommendations for Interventional Trials (SPIRIT) (Chan et al., 2013), see Supplementary Table 1 for Spirit Checklist. The trial will be conducted according to the Consolidated Standards of Reporting Trials (CONSORT) of Electronic and Mobile HEalth Applications and onLine TeleHealth (CONSORT E-HEALTH) statement (Eysenbach, 2011), CONSORT Social and Psychological Interventions (CONSORT-SPI) (Montgomery et al., 2018) guidelines and CONSORT extension for the reporting of non-inferiority trials (Piaggio et al., 2012). The trial has been registered on the Australian New Zealand Clinical Trials Registry (ANZCTRN2619001602112). Refer to Appendix I for more details.

The study will commence immediately following ethics approval and recruitment of participants will continue for ~2 years. The overall duration of the study will be 3 years to allow for those in the wait list control condition to complete the intervention and the follow up assessment, and for data analysis and dissemination of results. To test the efficacy of the intervention, the study will test the following hypotheses:

1. ACT-Adjust intervention: Participants in the active treatment regardless of delivery (FtF vs. VC) will demonstrate increased psychological flexibility and decreased psychological distress following the intervention, compared to those in the control group (waitlist).
2. eHealth: There will be no differences in primary and secondary outcomes between participants who receive the ACT-Adjust intervention by FtF compared to those who receive it via VC.
3. Sustained treatment effects: Participants in the active treatment condition, ACT-Adjust (VC and FtF), will maintain their gains three months post intervention.

### Participants and Settings

The study will recruit participants with a moderate to severe TBI through the community outpatients service Brain Injury Rehabilitation Program, NSW, Australia (BIRP) (Tate et al., 2004) and through icare, NSW. The BIRP is a network of multidisciplinary team-based, community outpatient services located in both regional and metropolitan areas of the state of NSW, Australia providing brain injury rehabilitation to individuals with a traumatic brain injury. The BIRP service is overseen by Rehabilitation physicians and a client centered approach to rehabilitation is undertaken. icare is a NSW state government organization that provides funding and case management services to individuals who have incurred a catastrophic injury (TBI, spinal cord and multiple limb amputations) as a result of a motor vehicle accident.

### Eligibility Criteria

Inclusion criteria will comprise: (i) having sustained a severe TBI (post-traumatic amnesia =>7 days) after 18 years of age; (ii) being between 18 and 65 years old; (iii) <5 years post-injury; (iv) having sufficient cognitive-linguistic capacity to complete self-report measures and participate in the program; and (v) reporting a clinically significant level of psychological distress (Depression >13, Anxiety >9, and Stress >18; Depression Anxiety Stress Scales 21-item; DASS-21) (Lovibond and Lovibond, 1995). Exclusion criteria are: (i) having a severe psychiatric illness, including psychotic disorder or substance addiction as determined by the medical file, self-report or consultation with the rehabilitation team; (ii) currently receiving a psychological intervention; and (iii) living >1-h travel distance from a psychologist able to deliver the intervention, to ensure access to a psychologist regardless of whether the therapy is delivered FtF or by VC.

### Recruitment Process

Screening will be conducted of individuals within the BIRP and icare services to identify prospective participants for the clinical trial. The following processes will be followed to recruit participants from the respective services:

BIRP: Health professionals employed by NSW Health at each BIRP location will review current clients of the service to assess suitability for the study. Researchers will contact staff at each BIRP to prompt regular reviews of the services' caseloads throughout the duration of the trial. Participants will be initially approached by their contact at the BIRP service either in person or by telephone (usually their case manager) and consent for the research team to contact the participant by phone will be sought. Following verbal consent, a member of the research team will contact participants and provide the participant information sheet and consent form (screen) by mail or email.

icare: Researchers will give a series of presentations to icare teams across NSW to promote the trial and recruitment of participants. Potential clients will be identified by icare case managers by one of two methods:(a) if they score <3 on either items 5 (*How much do you enjoy life?*) or 6 (*To what extent do you feel your life to be meaningful?*) or >3 on item 26 (*How often do you have negative feelings such as blue mood, despair, anxiety, depression?*) on the WHO Quality of Life—BREF measure (WHOQOL Group, 1998), which is administered as routine clinical practice by icare; or (b) by their knowledge of the participant's current situation. The icare staff member will approach the potential participant by telephone for possible involvement in the research and seek consent for the research team to contact the participant directly. Following verbal consent, a member of the research team will contact the participant by phone and provide the participant information sheet and consent form (screen) by mail or email.

### Randomization

The randomization process was performed by a researcher independent of the research team. A computer generated randomization list was generated for each phase of the study using online randomization software, Sealed Envelope (Sealed Envelope Ltd, 2019). The initial randomization had a ratio of 2:3:2 (FtF:WL:VC), with a permuted block size of seven. Following Time 2 Data Collection (13 weeks after Baseline/Time 1), wait list control participants will be randomized to the ACT-Adjust intervention on a ratio of 1:1 (FtF:VC), using a permuted block size of four. The results for both phases were placed in sequentially numbered opaque and sealed envelopes. Following completion of a participant's baseline assessment, the Research Assistant will open the next sequential randomized envelope available to determine the participant's assigned condition. The following allocations are required: ACT-Adjust face-to-face *n* = 16 participants; ACT-Adjust eHealth *n* = 16 participants; Waitlist control *n* = 24 participants.

### Treatment Conditions

All participants will receive their intervention in conjunction with constrained usual care (Freedland et al., 2011). Usual care may involve prescribed components of multidisciplinary interventions (e.g., social work, physiotherapy, occupational therapy, case management, speech therapy, diversional therapy, and/or vocational assistance) except for clinical psychology, as well as regular medical reviews by a rehabilitation physician and/or general practitioner. Participants who persistently report extremely high levels of psychological distress (DASS-21 subscales in the extremely severe range, as measured at the beginning of each session) or express suicidal intent during therapy, will be removed from the study and offered immediate psychological treatment. In addition, the treating clinician will assess participants' ability to attend to the session material whether from cognitive impairment or fatigue and make necessary treatment adjustments with regard to rest breaks and/or rescheduling sessions.

#### Wait List Control Condition

The wait list control group will receive constrained usual care as discussed above excluding clinical psychology. They will be engaged in their prescribed rehabilitation activities for a period of 13 weeks and then complete the Time 2 assessment (post), at which stage they will be randomized to receive either ACT-Adjust FtF or via VC.

#### Intervention: ACT-Adjust 2.0

ACT-Adjust 2.0 is a modified version of the original ACT-Adjust (Whiting et al., 2020), to enable individual delivery (as opposed to group delivery) by FtF and by VC and to overcome identified limitations of the previous treatment protocol (Whiting et al., 2020). These were the length of some sessions, difficulties assessing committed action, the complexity of some metaphors for individuals with a TBI and sustainability of treatment gains. The process of modifying ACT-Adjust, involved consultations with a highly experienced panel of clinical psychologists, with expertise in TBI and ACT and/or VC, who reviewed the original ACT-Adjust treatment protocol and made recommendations for changes. Modifications included reducing the length of some sessions and increasing the number of sessions provided from seven to nine sessions resulting in increased dosage. The original Session 4 was divided into two sessions to separate the observing self/self as context from present moment awareness/mindfulness (now Session 5). Committed action (now Session 7) included the addition of the Goal Attainment Scale (GAS) (Turner-Stokes, 2009) to systematically measure behavioral changes (committed action). The GAS is then reviewed in Sessions 8 and 9 to monitor committed action progress and Session 9 is an additional session to reduce risk of relapse. Additional instructions were also provided in the manual to aid other clinicians in delivering the intervention and a summary of each session's goals was provided at the beginning of each session.

The shortened session times (~60 min) is proposed to assist in reducing fatigue and improving attentional processes for participants with cognitive impairments. The additional relapse prevention session (Session 9), aims to improve sustainability of treatment gains which were not achieved in the original ACT-Adjust clinical trial (Whiting et al., 2020). Metaphors were substituted to be VC compatible and more concrete (e.g., Chinese Finger Trap) enhancing participants' comprehension of the session focus. Further strategies to compensate for participants' cognitive impairments and fatigue after TBI translated from the original ACT-Adjust trial, included reviewing and repeating session material at the beginning of the following session, and provision of a hardcopy of the participant workbook printed in size 14 font, with a Flesch Readability Ease rating of 76.4 and Flesch-Kincaid Grade Level of 4.8. In addition, the therapist facilitates completion of workbook exercises, and session specific text message reminders are provided to facilitate completion of between-session home tasks, and for fatigue management, sessions were scheduled at a time favored by the participant, breaks were provided if required and sessions rescheduled if participants felt unable to attend. A summary of the program content is available in Table 1. The intervention will be delivered throughout New South Wales, Australia, by authors DW, MM, and JW and other trained mental health professionals recruited to the project, using the ACT-Adjust 2.0 Therapist Manual.

**Table 1:** Summary of the ACT-Adjust 2.0 program.

**Session no**.	**Content**
1	**Confronting the Agenda** Introduction to the program—Guidelines and boundaries, reasons to see the psychologist, aims of ACT-Adjust, program outline Administer measures Exercise: Mindfulness activity (Mindfulness of the breath, 2 min) Confronting the agenda—identifying individual issues, workability, breathing, mindfulness exercise Home task—Introduce concept of home tasks, reasons for home tasks and home task contract, home task rating sheet Conclusion—Brief summary of the session
2	**Control is the Problem** Administer measures Exercise: Mindfulness activity (Observing thoughts, 3 min) Session introduction—Review of home task, review of previous session (workability, concept of control) Control is the problem—Normalcy of control, human suffering Exercises: Chocolate cake, Chinese finger trap exercise, Let suffering get close Home tasks—Session 2 home task, home task rating sheet Conclusion—Brief summary of the session
3	**Acceptance and Defusion** Administer measures Exercise: Mindfulness activity (Body scan, 5 min) Session introduction—Review of home task, review of previous session (normalcy of control, human suffering) Cognitive Fusion Exercises: Milk milk milk, Physicalise the thought Home tasks—Session 3 home task, home task rating sheet Conclusion—Brief summary of the session
4	**The Observing Self** Administer measures Exercise: Mindfulness activity (Mindfulness of the breath, 2 min) Session introduction—Review of home task, review of previous session (Defusion—milk milk milk, physicalise the thought) The contextualized self Exercises: Passengers on the bus, Observer Chessboard metaphor Home tasks—Session 4 home task, home task rating sheet Conclusion—Brief summary of the session
5	**Present Moment Awareness** Administer measures Exercise: Mindfulness activity (Observing thoughts, 3 min) Session introduction—Review of home task, review of previous session (self as context, passengers on a bus, chessboard metaphor) Mindfulness education Exercises: Mindfully walking, Eating a sultana Home tasks—Session 5 home task, home task rating sheet Conclusion—Brief summary of the session
6	**Values** Administer measures Exercise: Mindfulness activity (Gratitude, 5 min) Session introduction—Review of home task, review of previous session (mindfulness education, eating a sultana) Values Exercises: Values card sort, Values identification, Significant birthday exercise Home tasks—Session 6 home task, home task rating sheet Conclusion—Brief summary of the session
7	**Values and Committed Action** Administer measures
	Exercise: Mindfulness activity (Body scan, 10 min) Session introduction—Review of home task, review of previous session (values) Setting goals Goal attainment scale (GAS) Home tasks—Session 7 home task, home task rating sheet Conclusion—Brief summary of the session
8	**Review** Administer measures Exercise: Mindfulness activity (Body scan, 8 min) Session introduction—Review of home task, review of course Goal attainment scale (GAS) Exercise: Leaves on a stream Home tasks—Session 8 home task, home task rating sheet Conclusion—Brief summary of the session
9	**Review and Relapse Prevention** Administer measures Exercise: Mindfulness activity (Body scan, 10 min) Review and score GAS Review of program Forward planning and where to next

Where possible, all therapy sessions (both FtF and VC) will be delivered to participants in their homes. Where participants do not have access to a safe, private space in their home, an alternative local site will be arranged by the research team. To facilitate session attendance and home task completion, an automated text messaging service, Appointfix (Freedland et al., 2011), will be used to send appointment reminders to participants the day before each session, and personalized, session-specific home task reminders 2 days after Sessions 1–9, and additional reminders 1 week following Session 7, and 2 weeks following Session 8. See Table 2 for examples of text message templates.

**Table 2:** Automated text message reminders for session attendance and home task completion.

**Message type**	**Content**
Appointment reminder	A reminder about your ACT-Adjust appointment on [Date] at [Start time]. If you are feeling unwell and need to reschedule, please contact <Phone number>.
Home task reminders	[Client's first name], it was great to see you this week for your first session. Don't forget to practice the mindfulness activity and monitor any distressing events over the next week. See you at the next session. [Client's first name], well done on completing Session 2. Don't forget to carry your valued activity card with you this week. See you at the next session.
	[Client's first name], we hope you found this week's session helpful. Remember to practice thinking about your distressing thoughts at least 3 times over the next week. See you at the next session. Well done on completing Session 7, [Client's first name]! Remember to listen to the mindfulness recording at least twice this week and keep working toward your goals from the session.

Each participant enrolled in the trial will receive a Therapy Kit comprising a hardcopy of the ACT-Adjust 2.0 Client Workbook and individually wrapped materials for Session 2 (Chinese finger trap), Session 5 (packet of sultanas), and Session 6 (values card pack). All participants will complete the ACT-Adjust 2.0 program (Table 1), which is comprised of 9 × 1.5 h sessions. The week prior to commencing the program (and before randomization occurs), participants will meet with the treating psychologist in person to complete Baseline assessments. Sessions 1–7 will occur on a weekly basis, and a 2-week break will be placed between Sessions 7 and 8, and a 4-week break between Sessions 8 and 9. If required, sessions may be rescheduled by arrangement between the participant and treating psychologist, and will be rescheduled as soon after the original session as is feasible. Following the completion of Session 9, participants will meet with an independent blinded assessor to complete the Time 2 assessment measures (within 1 week of treatment completions). Three months after completing Session 9, participants will meet again with an independent blinded assessor to complete Time 3 assessment measures.

##### Face to Face (FtF) Condition

Participants randomized to the FtF condition will complete the ACT-Adjust intervention in their home, or, if this is not appropriate, at a location accessible for both the participant and the psychologist delivering the intervention.

##### Video-Consulting (VC) Condition

Participants allocated to the video-consulting condition will complete the ACT-Adjust intervention online using the HealthDirect Video Call program (Coviu, 2020) and will be provided with additional equipment to use for the duration of the intervention to facilitate and standardize the telepsychology delivery. Items provided to each participant in this condition include 1 × Samsung Galaxy 10.1 Tablet device and charging equipment with a prepaid data SIM (4G network if available), and one wired headset.

Once randomized to the VC condition, the Research Assistant will schedule a technology home visit with the participant to deliver the Therapy Kit and VC equipment, provide training on using the technology and complete a test run using the VC platform. An appropriate space within the home environment will be established to allow both safety and privacy for the participant while engaging in the intervention. A test of the internet upload and download speeds will also be conducted and recorded by the Research Assistant at the site. The tablet devices have been set up with access restrictions so only the HealthDirect Video Call platform is accessible, with all other icons removed from the home screen to simplify the display. Upon completion of the intervention, and before post-treatment assessment, VC equipment will be collected from the participant by the Research Assistant.

### Treatment Fidelity

To assess treatment adherence, all therapy sessions will be audio recorded by therapists using an app on their mobile phone. These audio files will be uploaded onto the secure servers of South Western Sydney Local Health District (SWSLHD) and then deleted from the therapist's device. The electronic files will be password protected and saved with the unique identifier for each participant. On completion of the study 15% of sessions (pragmatic selection to ensure reviews are completed for all sessions and all therapists) will be reviewed by an independent psychologist skilled in ACT to ensure adherence to the ACT-Adjust protocol. This will involve using both a checklist to ensure the therapist covers all aspects of the ACT-Adjust program and using the Acceptance and Commitment Therapy Fidelity Measure (ACT-FM) (O'Neill et al., 2019). The ACT-FM is a 25-item measure which assesses whether the therapist's behavior is consistent or inconsistent with ACT principles using a four point scale where 0 = “*The behavior never occurred*,” 1 = “*Therapist rarely enacts this behavior*,” 2 = “*Therapist sometimes enacts this behavior*,” and 3 = “*Therapist consistently enacts this behavior*.”

### Measures

Measures will be administered at baseline and for sessional measures, by the psychologist providing the intervention. Post treatment measures will be undertaken by a blinded assessor (see section Blinding for details about the blinded assessor), all assessors will be provided training in administration of the measures by DW to ensure consistency in administration. If required, the measures can be read out to the participant by the assessor to assist with understanding and a separate sheet with the measure's Likert response scales will be provided to the participant in larger font to compensate for vision, attentional/cognitive and reading deficits.

#### Primary Outcome Measures

*Psychological distress*—*Depression Anxiety Stress Scales* −*21 (DASS-21)* (Lovibond and Lovibond, 1995) is a 21-item questionnaire which assesses the constructs of depression, anxiety and stress over a seven-day period. Clinically significant levels of distress on the DASS-21 (87th percentile and above) are defined by the following scores on each subscale; (i) Depression >13, (ii) Anxiety >9, and (iii) Stress >18. The DASS-21 is commonly used in clinical practice within Australia and includes the broader psychological component of stress. The existing factor structure has been replicated in samples with a moderate to severe TBI and internal consistency of the subscales was very good (Cronbach's α > 0.80) (Randall et al., 2017).

*Psychological flexibility*—Acceptance and Action Questionnaire—Acquired Brain Injury (AAQ-ABI) (Whiting et al., 2015) is a nine-item self-report measure scored on a five-point Likert scale with a range of 0–36, with higher scores indicating greater psychological flexibility. Items assess psychological flexibility around the thoughts, feelings and behaviors that may arise after incurring a brain injury, e.g., Item three, “*I stop doing things when I feel scared about my brain injury*.” The measure has sound psychometric properties (e.g., Cronbach's α = 0.89) (Whiting et al., 2015).

#### Secondary Outcome Measures

*Quality of Life—Quality of Life after Brain Injury (QOLIBRI)* (Von Steinbüchel et al., 2010) is a health-related quality-of-life instrument specifically developed for traumatic brain injury (TBI). It has 37 items across six subscales scales; (i) cognition, (ii) self, (iii) daily life and autonomy, (iv) social relationships (v) emotions, and (vi) physical problems. The first four subscales are rated on a five-point Likert scale. It takes around 7–10 min to complete and has robust psychometrics with a Cronbach's α of 0.95 previously reported for the total score (Von Steinbüchel et al., 2010).

*Values identification—Survey of Life Principles (SLP) Version 2.2* is a card sorting task (Ciarrochi, 2009) and will serve a dual role in the study (i) measuring values importance and success; and (ii) during the intervention it is used to assist participants in values identification. The SLP has 60 items reflecting life principles (values) across various domains (e.g., “*acting with courage*,” “*designing things*”). Respondents allocate each principle to one of three categories; (1) not very important; (2) moderate importance; and (3) highest importance. From the highest importance category, respondents select their top 10 and rate them using a five-point Likert scale (0 = “not very” to 4 = “extremely”) on (1) How important was the value (Importance) and (2) How consistently are you acting in accordance with your value (Success). SLP scores of value importance and success have previously demonstrated good internal consistency (Cronbach's α = 0.79–0.97) (Ciarrochi, 2009).

*Committed Action—Engaged Living Scale (ELS)* (Trompetter et al., 2013) is a 16-item self-report measure which assesses an individual's engaged response style as conceptualized in ACT, e.g., Item eight, “*I believe that my values are really reflected in my behavior*.” The ELS uses a five-point Likert scale where 1 = “*completely disagree*” and 5 = “*completely agree*” and a score range (total score) of 16–80. It has been validated in chronic pain research using ACT and provides a better understanding of the mechanisms underlying change in ACT. It has two subscales assessing Valued Living (10 items) and Life Fulfillment (six items) and internal reliability on both subscales and the total scale is good (Valued Living, α = 0.86, Life Fulfillment, α = 0.87, total score ELS, α = 0.90) (Trompetter et al., 2013).

*Satisfaction with Life Scale (SWLS)* (Diener et al., 1985) is a five item, seven-point Likert scale measuring global satisfaction with life and is considered to assess a cognitive component of overall well-being. Higher scores are indicative of greater satisfaction with life. It has been used in several studies with TBI clients with good internal consistency, Cronbach alpha of 0.78 (Kreitzer et al., 2019).

#### Baseline Only Measures

*Repeatable Battery for the Assessment of Neuropsychological Status (RBANS)* (Randolph, 1998) will be used to provide an assessment of cognitive ability and will be administered at baseline only. The RBANS is a brief neurocognitive battery measuring immediate and delayed memory, attention, language, and visuospatial skills and has been found to be suitable for assessing cognitive function after TBI (Randolph, 1998). The RBANS requires approximately 25 min for administration and is broadly used for clinical diagnostic purposes to establish neurocognitive status.

*Project specific demographic questionnaire*—to collect demographic information regarding age, gender, injury circumstances and both premorbid and post injury functioning about work and relationships.

#### Session Measures

##### Pre–session

These measures are used for clinical purposes to assess the participant's emotional functioning on a weekly basis and include the DASS-21 and the AAQ-ABI which are described above.

##### Post-session–Clinical

*Session Rating Scale V. 3.0 (SRS)* (Duncan et al., 2003)—to assess therapeutic alliance between the participant and the therapist (both active treatment arms). The SRS is a short four-item measure to rate the experience of the treatment session from the perspective of the client. It covers the therapeutic relationship, goals and topics, the approach of the therapist and an overall session rating.

*Home Task Rating Sheet*—A measure of homework completion and adherence developed in the original ACT-Adjust study (Whiting et al., 2020). After the home task has been discussed with the participant, they are asked to rate how confident and how motivated they are to complete the home task using a five-point Likert scale of 1 = “Not at all” to 5 = “Totally.” At the beginning of the following session, after home tasks have been reviewed and discussed, participants are asked to rate their satisfaction with their home task completion on the same five-point scale. Any barriers to completing the task are selected from a tick list, e.g., “forgot,” “too busy,” discussed and any alternative reasons for non-completion of home tasks are noted by the therapist.

##### Post–session–Technical

These measures will be used to assess the feasibility of the video consulting intervention.

*VC Session Evaluation—Client* (project specific measure—video consulting only) relevant items were selected from the patient session review measure from the Agency of Clinical Innovation (ACI) telehealth guidelines (Freedland et al., 2011) and are included along with two items evaluating the picture and sound quality of the video link.

*VC Session Evaluation—Therapist* (project specific measure—video consulting only), has the same items as the client version but with more detail about the quality of the video link. It will be used to assess the quality of each session from the therapist's perspective and record any video or audio dropouts. A schedule of measure administration is provided in Table 3.

**Table 3:** SPIRIT schedule of enrolment, interventions, and assessments for ACT-adjust.

			**Study period**								
			**Enrolment**	**Allocation**	**Post-allocation**						
					**Intervention**				**Evaluation**		
Time Points			T1	T1a	T1b	Weeks 1–7	Week 9	Week 13	T2	T3	T4
					VC training	Session 1–7	Session 8	Session 9			
Enrolment		Eligibility screen	X								
		Informed consent	X								
		Randomization		X							
Interventions		ACT-Adjust FtF				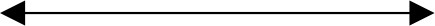					
		ACT-Adjust VC			X	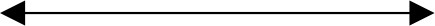					
		Wait list Control				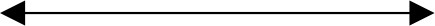					
Assessments	Baseline measures	Demographic data	X								
		RBANS	X								
	Primary Outcome measures	DASS-21	X			X^2^	X^2^	X^2^	X	X	X
		AAQ-ABI	X			X^2^	X^2^	X^2^	X	X	X
	Secondary Outcome measures	QOLIBRI	X						X	X	X
		SLP	X						X	X	X
		ELS	X						X	X	X
		SWLS	X						X	X	X
	Other measures	HTRS^1^				X^2, 3^	X^2, 3^	X^2, 3^			
		SRS V.3				X^3^	X^3^	X^3^			
		VCSE-Client				X^3^	X^3^	X^3^			
		VCSE-Therapist				X^3^	X^3^	X^3^			

1 *Items 1-2 administered at end of the session and Item 3 at the beginning of the following of session*;

2 *Measure administered at the beginning of the treatment session*;

3 *Measure administered at the conclusion of the treatment session. AAQ-ABI, acceptance and action questionnaire-acquired brain injury; DASS-21, depression anxiety stress scales −21; ELS, enhanced living scale; HTRS, home task rating scale; QOLIBRI, quality of life after brain injury; RBANS, repeatable battery for the assessment of neuropsychological status; SWSL, satisfaction with life scale; SRS V.3, session rating scale version 3; VCSE, video consulting session evaluation*.

### Sample Size and Power Calculations

Based on a previous pilot RCT of the ACT-Adjust program (Whiting et al., 2020), sample size calculations on the primary outcome measure (AAQ-ABI) indicate a total sample size of 56 (ES Partial η^2^ = 0.17, Power = 0.80), is required to establish a significant interaction effect from pre to post intervention. A previous RCT for a psychological intervention conducted at the Liverpool Hospital, Brain Injury Rehabilitation Unit screened *n* = 90 participants with *n* = 17 meeting study criteria and agreeing to participate in the trial (Simpson et al., 2011). Using this ratio, it is anticipated ~*n* = 297 participants will need to be screened to recruit the required sample for the current trial.

### Informed Consent

During the consent process, all participants will be informed as to the purpose of the research, any potential risks or benefits and processes to withdraw from the study and provided with the Participant Information Sheet (see Supplementary File 2). By definition, clients who have the cognitive capacity to participate in the ACT-Adjust program, will have sufficient cognitive capacity to provide written informed consent. Participants' capacity to provide informed consent will be determined by a review of any available neuropsychological assessment and in consultation with their treating health professionals including their Rehabilitation Specialist. Consent will be fully explained to the participants in simple language and they will be given the opportunity to have a family member present during the consent process. Language will be used at an appropriate level for participants' cognitive impairment and extra time will be provided for participants to process and think about the research. All consent for the trial will be undertaken by the Chief Investigator (DW).

### Blinding

Psychologists conducting baseline assessments (Time 1) will be blinded to participant treatment allocation, as participants will be randomized after the baseline assessment (Time 1). Blinding of the treating psychologists providing the intervention is not possible, however an independent assessor will be used to complete the post-treatment (Time 2) and 3-month follow-up assessments (Time 3) and will be blinded to the condition. The success of blinding will be assessed by having the independent assessor (i) indicate which condition they think the participant was randomized to (FtF, WL or VC) and (ii) indicate if the participant disclosed their treatment condition. Data analysis will be undertaken by a statistician, from the Ingham Institute for Applied Medical Research but not directly associated with the individual research group, who will be blinded to the treatment conditions. It is not anticipated unblinding will be required for the independent assessor or statistician and therefore is not permissible in this study.

### Data Analysis

Data will be entered into SPSS statistics software (IBM Corp, 2019), reviewed for missing data and then screened for normality on all outcome measures and for each treatment group. Prior to the main analysis, between groups comparisons will be undertaken to test for equivalency of the groups using chi-square and *t*-tests. Where participants withdraw or are not contactable for the follow up assessment, the last recorded measures will be carried forward to remaining assessment points for data analysis. Primary analysis will be implemented on an Intention-to-Treat basis, using hierarchical linear mixed models to study the differential effects of each treatment condition. For the post-treatment analyses between the three treatment conditions, analysis will focus on linear time effects, treatment conditions and interactions. Cohen's effect size will be calculated for all analyses. Analyses will focus on the estimated mean differences relative to pre-treatment levels on primary and secondary outcome measures (see Table 3). For the longer-term treatment effects, the non-inferiority of the face-to-face and video consulting will be assessed at the 3-month follow-up.

## Discussion

Results from this project will have implications for how barriers to psychological treatment access can be overcome amongst individuals with a TBI, living in remote and regional areas of Australia. Improved accessibility will be provided to these individuals by psychologists appropriately trained in using evidence-based psychological treatment. Furthermore, it will extend the current evidence base of the ACT-Adjust psychological treatment of mental health issues in individuals with a TBI. Finally, it will also have implications for telepsychology, that is, psychological treatments delivered by video consulting.

The trial aims to address mental health issues for individuals with a moderate to severe TBI living across all areas of NSW, Australia with the aim of improving overall quality of life by reducing psychological distress and increasing psychological flexibility. This project is significant since it is the first phase II trial that tests the efficacy of an ACT program for civilians and the first trial using an eHealth modality (telepsychology by VC) with clients experiencing a TBI. The project will (i) contribute to the evidence base for Acceptance and Commitment Therapy (ACT), eHealth and treatment of mental health issues after a TBI, and (ii) assist in addressing access to psychological therapy for individuals who may have difficulty seeing a psychologist face to face due to geographical location or transportation limitations.

### Potential Strengths of the Study

The study has been designed to meet the CONSORT guidelines for non-inferiority trials, psychosocial interventions and eHealth delivery (Eysenbach, 2011; Piaggio et al., 2012; Montgomery et al., 2018). It builds on a previously published RCT using an adaptation of the ACT-Adjust program which indicated statistically and clinically significant reductions in depression and stress but with a smaller sample size (Whiting et al., 2020). The project is a multi-site study to enable recruitment across a wide geographical area and will recruit individuals living across the state of NSW, Australia. The ACT-Adjust intervention will be delivered by clinicians (e.g. clinical psychologists, social workers, etc.) with experience in both ACT and TBI.

### Potential Limitations of the Study

There are some potential limitations in the generalizability of findings to use of eHealth in wider clinical practice contexts. Specifically, this research supplies participants with equipment and internet data to ensure efficient and seamless telepsychology sessions are delivered by VC. Also, participants will be trained by the Research Assistant in using the VC platform prior to commencing treatment. This level of service is unlikely to be achieved in general clinical practice where clinicians will have to use existing client knowledge and resources including both software and hardware. The study is also limited to English speaking participants and those without current comorbid psychiatric symptoms, therefore there is limited generalizability to other culturally and linguistically diverse groups as well as to individuals with comorbidities.

This study meets the gold standard of clinical trial delivery to provide additional evidence of ACT with individuals who have a TBI. It will also contribute to the research base on feasibility and effectiveness of eHealth for people with TBI.

## Ethics and Dissemination

### Ethical Considerations

Ethical approval for the study was granted by the South Western Sydney Local Health District Human Research Ethics Committee (2019/ETH13152), and local site specific assessments were authorized across trial sites (Liverpool Brain Injury Rehabilitation Unit: 2019/STE16924; Bathurst Health Service: 2019/STE17981; Illawarra Brain Injury Service: 2019/STE17982). Any protocol amendments will be reviewed by the steering committee and then be submitted for approval to the relevant ethics committee, for example, the addition of more recruitment sites. These amendments will also be submitted for approval to the clinical trial registry, ANZCTR.

Confidentiality for participants will be ensured as all measures administered will be deidentified using a unique identifier. The unique identifier will be linked to the participant's name and this personal information stored separately in a password protected spreadsheet located on a secure server at SWSLHD, accessed by only the co-ordinating Chief Investigator (DW) and Research Assistant (SC). Consent forms (paper versions) will be stored separately from measures administered (see below for data storage).

Any incidents of harm will be recorded in a secure file and ongoing management of participants will be undertaken by the psychologist delivering the intervention who will record participants' weekly distress using a self-report measure (DASS-21). In addition, a suicide risk assessment is included in the treatment protocol in the first week of treatment. Some participants will be undertaking other rehabilitation treatment and all participants will have a nominated treating doctor to provide additional support. Participants will be given phone numbers and contact details of support systems between sessions. This will ensure adequate monitoring of participants will occur during the trial. Post-trial care will be maintained by the rehabilitation team at the BIRP or icare treatment team. It is not anticipated any harm will occur from participants receiving this treatment, as both clinical and statistically significant improvements in mood were achieved when the intervention was delivered in previous research (Whiting et al., 2020).

### Data Storage and Retention

While the study is ongoing, paper-based participant data will be stored in locked filing cabinets on Level 2 at the Ingham Institute for Applied Medical Research. The Ingham Institute is a secure facility with swipe card access and all visitors are verified and accompanied by a staff member whilst on the premises. Data stored electronically will be password protected and kept on secure servers at the SWSLHD, which is backed up daily. Only investigators outlined within this protocol will have access to the data and the final trial dataset.

### Dissemination

Dissemination of the study will be undertaken by presenting results at relevant conferences, publishing results in peer reviewed journals and in a final report provided to the icare Foundation. Participants who indicate on the consent form they would like feedback on the study, will be provided with a brief report written in plain language by post. All researchers involved in the project will have the opportunity to be involved in any publications which will be written by the primary researchers (DW, GS, and SC). Ongoing access to the data for later inclusion in metanalyses and systematic reviews will be subject to application and consideration by the primary researchers. The ACT-Adjust 2.0 treatment manual will be available to clinicians and researchers in PDF format by direct application made to the primary researchers.

## Ethics Statement

The studies involving human participants were reviewed and approved by South Western Sydney Local Health District Human Research Ethics Committee. The patients/participants provided their written informed consent to participate in this study.

## Author Contributions

DW, GS, and FD were involved in the development of this study protocol. DW and GS contributed to the writing of the funding application to icare. This manuscript was drafted by DW, GS, and SC and has been reviewed, edited, and the final version approved by all listed authors.

## Conflict of Interest

The authors declare that the research was conducted in the absence of any commercial or financial relationships that could be construed as a potential conflict of interest.

## Abbreviations

AAQ-ABI: acceptance and action questionnaire—acquired brain injury
ACT: acceptance and commitment therapy
ACT-FM: acceptance and commitment therapy fidelity measure
ANZCTR: Australian New Zealand clinical trials registry
BIRP: brain injury rehabilitation program, NSW, Australia
CONSORT: consolidated standards of reporting trials
DASS-21: depression anxiety stress scales−21
ELS: enhanced living scale
FtF: face-to-face
HTRS: home task rating scale
NSW: New South Wales
RCT: randomized controlled trial
SPIRIT: standard protocol items as recommended for interventional trials
SWSLHD: South Western Sydney local health district
QOLIBRI: quality of life after brain injury
RBANS: repeatable battery for the assessment of neuropsychological status
SWSL: satisfaction with life scale
SRS V.3: session rating scale version 3
TBI: traumatic brain injury
VC: video consulting
VCSE: video consulting session evaluation
WL: waiting list.

## Appendix I: Items From the World Health Organization Trial Registration Data Set

**Table 1:**

**Data category**	**Information**
Primary registry and trial identifying number	ANZCTR.org.au ACTRN12619001602112P
Date of registration in primary registry	11 November, 2019
Secondary identifying numbers	U1111-1241-7053
Source(s) of monetary or material support	icare NSW
Primary sponsor	South Western Sydney Local Health District (SWSLHD)
Secondary sponsor(s)	N/A
Contact for public queries	DW (diane.whiting@health.nsw.gov.au)
Contact for scientific queries	DW, Ingham Institute for Applied Medical Research, Liverpool, NSW, Australia
Public title	Treating psychological distress after traumatic brain injury (TBI) either by face to face or video consulting.
Scientific title	Evaluation and feasibility of eHealth for individuals with a traumatic brain injury (TBI) experiencing psychological distress: ACT-Adjust via video consulting.
Countries of recruitment	Australia
Health condition(s) or problem(s) studied	Depression Traumatic brain injury (TBI) Anxiety Stress
Intervention(s)Key inclusion and exclusion criteria	ACT-Adjust: Videoconferencing Active Comparator: ACT-Adjust: Face-to-Face Control comparator: Waiting list
	Sexes eligible for study: both
	Accepts healthy volunteers: no
	Inclusion criteria: (i) having sustained a severe TBI (post-traumatic amnesia) =>7 days after 18 years of age; (ii) aged between 18 and 65 years; (iii) sufficient cognitive and linguistic capacity to complete self-report measures and participate in the program; (iv) reporting a clinically significant level of psychological distress (Depression>13, Anxiety>9, and Stress>18; Depression Anxiety Stress Scales 21-item; DASS-21) Lovibond and Lovibond, 1995
	Exclusion criteria: (i) having a severe psychiatric illness, including psychotic disorder or substance addiction as determined by the medical file, self-report or consultation with the rehabilitation team; (ii) currently undergoing psychological intervention; and (iii) living >1-h travel distance from a psychologist able to deliver the intervention.
Study type	Interventional
	Allocation: randomized intervention model. Parallel assignment masking: blind (outcomes assessor; data analysis)
	Primary purpose: treatment
	Phase II
Date of first enrolment	March 2020
Target sample size	56
Recruitment status	Recruiting
Primary outcome(s)	Changes in psychological distress and psychological flexibility, as measured using the validated Depression Anxiety Stress Scales (DASS-21) and the validated Acceptance and Action Questionnaire—Acquired Brain Injury (AAQ-ABI). Timeframe: Baseline, at completion of therapy and 3 months following therapy intervention
Key secondary outcomes	Changes in: Quality of Life, as measured using the validated Quality of Life after Brain Injury (QOLIBRI); identified values (how consistently a person acts in accordance with these values), as measured using the Survey of Life Principles (SLP) Version 2.2; committed action (a client's engagement in the therapy process), as measured using the Engaged Living Scale (ELS); global measures of life satisfaction, as measured using the validated Satisfaction with Life Scale (SWLS); and perceived importance of identified values, using the Survey of Life Principles (SLP Version 2.2). Timeframe: Baseline, at completion of therapy intervention and 3 months post-intervention
