# Supplementary material for: Protocol for a Phase Two, Parallel Three-Armed Non-inferiority Randomized Controlled Trial of Acceptance and Commitment Therapy (ACT-Adjust) Comparing Face-to-Face and Video Conferencing Delivery to Individuals With Traumatic Brain Injury Experiencing Psychological Distress
Source: Front Psychol. 2021 Mar 8;12:652323. doi: 10.3389/fpsyg.2021.652323 (PMC7982655; doi:10.3389/fpsyg.2021.652323)
Supplement: Supplementary file 2 [file Table_2.docx]

*[Insert Site Logo]*

**Participant Information Sheet**

**Interventional Study** - *Adult providing own consent*

*[Insert site name]*

| **Title** | Evaluation and feasibility of eHealth for individuals with a traumatic brain injury (TBI) experiencing psychological distress: ACT-Adjust Video consulting |
| --- | --- |
| Short Title | ACT-Adjust VC-Trial |
| Protocol Number | Version 1 |
| Project Sponsor | South Western Sydney Local Health district (SWSLHD) |
| Coordinating Principal Investigator | Dr Diane Whiting |
| Co-Investigators | Professor Grahame Simpson  Professor Frank Deane |
| Associate Investigator(s) | Ms Sarah Chuah  Dr Michelle Maitz |
| **Location** | *[Location]* |

**Part 1 What does my participation involve?**

1. **Introduction**

ACT-Adjust is a tested psychological counselling program that aims to help people make a positive adjustment to living with a traumatic brain injury (TBI). The trial will try to find out if providing the ACT-Adjust program through the computer or tablet (video consulting) is the same as providing ACT-Adjust face to face.

You are invited to take part in the research project called ACT-Adjust VC Trial because of the answers that you gave in the surveys that were part of the Screening study (ACT-Adjust Screen).

This Participant Information Sheet tells you about the research project. It explains the tests and treatments involved. Knowing what is involved will help you decide if you want to take part in the research.

Please read this information carefully. Ask questions about anything that you don’t understand or want to know more about. Before deciding whether or not to take part, you might want to talk about it with a relative, friend or your local doctor.

Participation in this research is voluntary. If you don’t wish to take part, you don’t have to. You will receive the best possible care whether or not you take part.

If you decide you want to take part in the research project, you will be asked to sign the consent section. By signing it you are telling us that you:

- understand what you have read
- consent to take part in the research project
- consent to have the tests and treatments that are described
- consent to the use of your personal and health information as described.

You will be given a copy of this Participant Information Sheet to keep.

**2 What is the purpose of this research?**

The ACT-Adjust program has helped people feel less stressed and depressed after their TBI when delivered face to face. The trial will try to find out if providing the ACT-Adjust program through video consulting is the same as providing ACT-Adjust face to face.

If the trial is a success, then it will improve access to psychological interventions for those people living in country areas of Australia. It can also help people in cities who find it hard to drive or catch buses/trains to get to see a psychologist.

This research has been initiated by Dr Diane Whiting and Professor Grahame Simpson. It is funded by a grant from icare and is being conducted by the Brain Injury Rehabilitation Research Group at the Ingham Institute for Applied Medical Research, Liverpool Sydney.

**3 What does participation in this research involve?**

1. Reading the trial Participant Information Sheet and signing the study Consent form;
2. Completing some surveys and cognitive test at up to four time points (Baseline and/or Pre-treatment, Post-treatment and follow-up); and
3. Receiving the ACT-Adjust program, as delivered by a trained psychologist with expertise in counselling people with TBI, either face to face or through video conferencing.

**4 What do I have to do?**

1. Read Participant Information Sheet and Sign the Consent form

Having read the Participant Information Sheet, if you agree to take part in the study, you will sign a consent form.

1. Baseline information

The next step will be to sit down with a psychologist and do some surveys and cognitive tests. This will take about 90 minutes of your time. These surveys and tests will give us important information about how you are feeling and thinking. A time can be arranged to do this assessment when it is most convenient for you. This can be either at your home or at your local brain injury service. Should there be a delay in beginning the treatment, further testing will be required immediately prior to starting treatment.

1. Allocated to treatment

You will be taking part in a randomised controlled research project. After the baseline information has been collected you will be randomly allocated to either one of two arms of the trial (face to face or video consulting) to receive the ACT-Adjust program.

Random allocation is like flipping a coin to decide which way you will receive the treatment. You have a one in two chance of being allocated to either receiving the ACT-Adjust program face to face or by your computer or tablet (video-conferencing).

We will not be able to treat everyone straight away, and so there will be a wait time for some participants before they can start the ACT-Adjust program.

1. Home technology visit

If you are allocated to receive the ACT-Adjust program via video-conferencing, a member of the research team will contact you and come to visit you to work out the best technology option for you to do the program from. This might be from your computer at home, or it might be from a room set up for video-conferencing at your local health service. If you don’t have a computer or internet access, we will lend you a tablet with internet access for the duration of the trial.

1. Doing the ACT-Adjust program

The ACT-Adjust program will run for nine sessions. The first 7 sessions will be conducted on a weekly basis. There will then be a 2 week break before session 8 is delivered. There will then be a four week break before doing the last session (session 9). Each session will run for about 1 hour.

At the beginning of each session, you will complete a brief survey about how you have been feeling and acting since the previous session. At the end of each session, there is a brief survey about how you liked the session. People receiving ACT-Adjust by video-conferencing also answer two questions about quality of the video and the sound.

1. Follow-up questionnaires

Once you have finished the last session of the ACT-Adjust program, a member of the research team will contact you by phone to complete some surveys. The same person will then contact you 3 months later to do the surveys one last time.

From the beginning of the program to the final assessment will take about 6 months. After the final assessment, you will be given the opportunity to continue with individual treatment provided by a mental health professional if you choose.

1. Taping the Act-Adjust sessions

All face to face sessions will be audio-taped and video sessions will be video-taped so that we are able to make sure the ACT-Adjust program is being delivered to you as it is set out in the manual. A sample of these tapes will be reviewed by a member of the research team who does not know the participants.

To maintain confidentiality, the facilitator will ask people not to provide any identifying information in the session such as age, address or last names. The tapes will be held in a secure place and the records erased after the statutory period for the maintenance of research records has expired.

**5 Other relevant information about the research project**

Overall we hope to have 56 people complete the treatment program over the next two years. We will be recruiting people with a TBI across all of NSW who are clients of icare, and/or NSW Health. The treatment will be delivered by a number of psychologists from both NSW Health services and those working in a private practice. This will involve up to three metropolitan and three regional hospitals.

This research project has been designed to make sure the researchers interpret the results in a fair and appropriate way. There are no additional costs associated with participating in this research project, nor will you be paid.

**6 Do I have to take part in this research project?**

Participation in any research project is voluntary. If you do not wish to take part, you don’t have to. If you decide to take part and later change your mind, you are free to withdraw from the project at any stage. If you do decide to take part, you will be given a Consent Form to sign and you will be given a copy of this sheet to keep.

Your decision whether to take part or not to take part, or to take part and then withdraw, will not affect your routine treatment, your relationship with those treating you or your relationship with [*Health Service]* or icare.

**7 What are the alternatives to participation?**

You do not have to take part in this research project to receive psychological treatment. Other options are available; these include seeing a psychologist independent of the study. Your case manager will discuss these options with you before you decide whether or not to take part in this research project. You can also discuss the options with your treating doctor.

**8 What are the possible benefits of taking part?**

We cannot guarantee or promise that you will receive any benefits from this research; however, possible benefits may include improved mood and reduced psychological distress.

**9 What are the possible risks and disadvantages of taking part?**

Some psychological treatments cause people to feel increased emotions or distress as they work through the treatment program. If you become upset or distressed as a result of your participation in the research, this will be addressed by the intervention being delivered.

If you feel this is insufficient and you are unable to continue with the research program, the study organiser will be able to arrange for counselling or other appropriate support. Any counselling or support will be provided by qualified staff who are not members of the research project team. This counselling will be provided free of charge.

**10 Can I have other treatments during this research project?**

Whilst you are participating in this research project, you may continue with any usual rehabilitation treatment, other programs as well as medication. Other treatment provided by a psychologist independent to the study is not permitted.

**11 What if I withdraw from this research project?**

If you decide to withdraw from the project, please notify a member of the research team before you withdraw, this will usually be the psychologist who is providing treatment. This notice will allow that person or the research supervisor to discuss alternative psychological treatment options for you.

If you do withdraw your consent during the research project, the study staff will not collect additional personal information from you, although personal information already collected will be retained to ensure that the results of the research project can be measured properly and to comply with law. You should be aware that data collected by the research team up to the time you withdraw will form part of the research project results. If you do not want the research team to do this, you must tell them before you join the research project.

**12 What happens when the research project ends?**

When the project is finished, you can continue to see a psychologist if you feel you need further assistance. The results of the study will be published and presented at conferences. You can access the results of the research from the icare web page.

**Part 2 How is the research project being conducted?**

**13 What will happen to information about me?**

By signing the consent form you consent to the relevant research staff collecting and using personal information about you for the research project. Any information obtained in connection with this research project that can identify you will remain confidential. All information collected will be deidentified (i.e. each person will be given a number and we will not use your name). Any information that is obtained in connection with this study and that can be identified with you will remain confidential and will be disclosed only with your permission or except as required by law.

Information about you may be obtained from your health records held at this and other health services for the purpose of this research such as your injury circumstances. By signing the consent form you agree to the study team accessing health records if they are relevant to your participation in this research project.

It is anticipated that the results of this research project will be published and/or presented in a variety of forums. In any publication and/or presentation, information will be provided in such a way that you cannot be identified, except with your permission. All information will be presented as group data without individual identification. In any publication, information will be provided in such a way that you cannot be identified.

In accordance with relevant Australian and/or NSW privacy and other relevant laws, you have the right to request access to your information collected and stored by the research team. You also have the right to request that any information with which you disagree be corrected. Please contact the study team member named at the end of this document if you would like to access your information.

Any information obtained for the purpose of this research project that can identify you will be treated as confidential and securely stored. It will be disclosed only with your permission, or as required by law.

**14 Complaints and compensation**

If you suffer any injuries or complications as a result of this research project, you should contact the study team as soon as possible and you will be assisted with arranging appropriate psychological treatment.

**15 Who is organising and funding the research?**

This research project is being conducted by Dr Diane Whiting and Professor Grahame Simpson. No member of the research team will receive a personal financial benefit from your involvement in this research project (other than their ordinary wages).

The research is being funded by icare, a state government instrumentality, and administered through the Ingham Institute for Applied Medical Research.

**16 Who has reviewed the research project?**

The original proposal for the project was forwarded to icare, who sent it out for external review, before making the decision to fund the project.

All research in Australia involving humans is reviewed by an independent group of people called a Human Research Ethics Committee (HREC). The ethical aspects of this research project have been approved by the HREC of South Western Sydney Local Health District (SWSLHD). This project will be carried out according to the *National Statement on Ethical Conduct in Human Research (2007)*. This statement has been developed to protect the interests of people who agree to participate in human research studies.

**17 Further information and who to contact**

If you would like any further information concerning this project you can contact any of the following people:

**Principal Researcher and** **Clinical contact person**

| Name | Dr Diane Whiting |
| --- | --- |
| Position | Project Manager / Clinical Psychologist |
| Telephone | 0423132447 |
| Email | diane.whiting@health.nsw.gov.au |

Other research team members:

| Name | Ms Sarah Chuah |
| --- | --- |
| Position | Research Assistant |
| Telephone | 02 8738 9051 |
| Email | sarah.chuah@health.nsw.gov.au |

For matters relating to research at the site at which you are participating, the details of the local site complaints person are:

*[Insert contact details]*

**Reviewing HREC approving this research** **and HREC Executive Officer details**

This study has been approved by the South Western Sydney Local Health District Human Research Ethics Committee. Any person with concerns or complaints about the conduct of this study should contact the Research and Ethics Office, Locked Bag 7103, LIVERPOOL BC NSW 1871 on 02 8738 8304 / fax 02 8738 8310 / email [research.support@health.nsw.gov.au](mailto:research.support@health.nsw.gov.au), website: <http://www.swslhd.nsw.gov.au/ethics/default.html> and quote [*Local project number*].

**Thank you for taking the time to consider this study.**

**If you wish to take part in it, please sign the attached consent form.**

**This information sheet is for you to keep.**
